# Effects of Aroclor 1254 on *In Vivo* Oocyte Maturation in the Mouse

**DOI:** 10.1371/journal.pone.0102064

**Published:** 2014-07-11

**Authors:** ShuZhen Liu, LiGang Jiang, XiaoQian Meng, XiaoYing Han, Dong Cheng, TianLiang Zhang, YiLiang Miao

**Affiliations:** 1 Key Laboratory of Animal Resistance Research, College of Life Science, Shandong Normal University, Jinan, China; 2 Center for Reproductive Medicine, Qilu Hospital of Shandong University, Jinan, China; 3 Department of Toxicology, Shandong Center for Disease Control and Prevention, Jinan, China; 4 Key Laboratory of Animal Genetics, Breeding, and Reproduction of Ministry of Education, College of Animal Science and Technology, Huazhong Agricultural University, Wuhan, China; Institute of Zoology, Chinese Academy of Sciences, China

## Abstract

Polychlorinated biphenyls (PCBs) are stable, lipophilic compounds that accumulate in the environment and in the food chain. Though some studies provided evidence that PCBs had adverse effects on reproductive function, most of these results were from *in vitro* models. Therefore we investigated the effect of Aroclor 1254 (a commercial PCBs mixture) treatments on *in vivo* maturation and developmental potential of mouse oocytes. In the present study, female ICR mice were treated with different doses (12.5, 25 and 50 mg/kg) of Aroclor 1254 (a commercial PCB mixture) once every 72 hours by intraperitoneal injection for 9 days. After three treatments of Aroclor 1254, the mice were superovulated to collect oocytes one day after the last exposure. The effects of Aroclor 1254 on oocyte maturation, fertilization, and preimplantation embryonic development were investigated. Immunofluorescence-stained oocytes were observed under a confocal microscope to assess the effects of Aroclor 1254 on spindle morphology. Parthenogenic activation and the incidence of cumulus apoptosis in cumulus-oocyte complexes were observed as well. Oocytes exposed to different doses of Aroclor 1254 *in vivo* were associated with a significant decrease in outgrowth potential, abnormal spindle configurations, and the inhibition of parthenogenetic activation of ovulated oocytes. Furthermore, the incidence of apoptosis in cumulus cells was increased after exposed to Aroclor 1254. These results may provide reference for the treatment of reproductive diseases such as infertility or miscarriage caused by environmental contaminants.

## Introduction

Polychlorinated biphenyls (PCBs) are environmental contaminants which often have endocrine disrupting activity. Since the 1970s, the use and production of PCBs have been banned in many countries due to the growing awareness of the toxicity of PCBs, so the environmental level of PCBs has declined significantly. However, as a result of chemical stability and strongly lipophilic nature, PCBs have slow degradation rates in the environment and tend to biomagnifying and bioaccumulation in lipid-rich tissues. These compounds had been detected around the world in remote areas [1], [2], wildlife refuges [3], dairy products [4], and human breast milk [5]. These chemicals have attracted widespread attention due to their persistence in the environment, their ability to concentrate in food chains, and their continuous detection in the food supply and drinking water. The influence of PCBs on humans is of concern because of universal exposure to PCBs through ingestion of contaminated food, and dermal contact of contaminated surfaces, and inhalation of contaminated air [1]. They also seemed to accumulate in organisms and then can cause endocrine disruption at environmentally relevant exposure levels [6]. An environmentally relevant PCB mixture affected oocyte maturation, fertilization and embryo development at doses that ranged between 0.001 and 1 mg/ml, the minimum effective dose (0.001 mg/ml) being approximately 10-fold lower than the average level detected in human follicular fluid [7], [8], [9].

The main effort of experimental and epidemiological studies so far has been to survey the effects of chronic exposure to PCBs [10], [11]. However, information on female reproductive toxicity induced by a short-term exposure to PCBs *in vivo* is not yet available. Furthermore, most data available on the effects of PCBs on mammalian oocytes were from *in vitro* models [12], [13]. As a consequence, the effects of brief exposure to PCBs on the *in vivo* maturation of oocytes, subsequent fertilization and embryo development have not been adequately studied. This information is needed because of the growing number of poisoning incidents due to accidental ingestion of seafood or cooking oil contaminated by PCBs. PCBs exist in the environment in the compounds of isomers and congeners with different numbers of chlorine atom substituted on different positions of the biphenyl moiety and organisms are rarely exposed to a single congener [14]. Therefore to get a comprehensive toxicological evaluation of PCBs, more accurate data on the effects of mixtures and single congeners are needed.

Aroclor 1254 was chosen because similar mixtures have been used in industry [15]. This mixture is a commercial PCB mixture containing 54% chlorine by weight and has been widely used in toxicity research. The purpose of the present study is to investigate the effect of Aroclor 1254 treatments on mouse oocyte maturation *in vivo* and its developmental potentiality.

## Materials and Methods

### Animals and chemicals

Animal care and use were conducted in accordance with the Animal Research Institute Committee guidelines of the Ethics Committee of Shandong Normal University, China. Mice were housed in a temperature-controlled room with proper darkness-light cycles, fed with a regular diet, and maintained under the care of the Laboratory Animal Unit, Shandong Normal University, China. The mice were killed by cervical dislocation. This study was specifically approved by the Committee of Animal Research Institute, Shandong Normal University, China.

Female ICR mice, 4–5 weeks old and body weight in 25–30 g, were provided by the Beijing HFK Bio-Technology Co. Ltd (Beijing, China). The mice were allowed to adapt for at least 7 days in the experimental conditions, and were then randomly assigned to four groups.

Aroclor 1254 was obtained from SUPELCO, USA. Pregnant mare serum gonadotropin (PMSG) and human chorionic gonadotropin (hCG) were purchased from the Ningbo Second Hormone Factory (China). All other chemicals used in the present study were obtained from Sigma Chemical Co., unless indicated otherwise

### Aroclor 1254 treatment

Aroclor 1254 dissolved in olive oil at the time of treatment, was administered to female mice by intraperitoneal injection once every 72 hours at dose levels of 12.5, 25 and 50 mg/kg b.w. (administration volume: 0.05 ml/10 g, 2.5 percent of the Aroclor 1254 LD50 for an ip injection to mice is 2000 mg/kg body weight) for three times. In parallel, females receiving a comparable volume of olive oil were used as negative controls. The mice were used to induce superovulation after treatment for three times.

The dose was on the basis of a paper published in *Environmental Health Perspectives* [16], which was reported that the rats were treated with 25 mg/kg Aroclor 1254 for three times. We calculated the amount of Aroclor 1254 in our study as followed: the highest dose of mouse  =  multiply 25 mg/kg by the Km factor (6) for a rat and then divide by the Km factor (3) for a mouse [17].

### Collection of oocytes

After one day of recovery, the mice were superovulated with an intraperitoneal injection of 10 IU PMSG followed by 10 IU hCG after 46–48 h. About 13–15 h after hCG injection, the mice were sacrificed via cervical dislocation and oocytes collected oocytes. Cumulus cells were removed by a brief hyaluronidase treatment [18]. These oocytes were then washed in PBS medium three times. Only oocytes with first polar bodies were used for parthenogenetic activation, and the observation of spindle morphology.

In addition, to evaluate the effects of Aroclor 1254 upon the surrounding granulosa cells, germinal vesicle stage oocytes attached with cumulus cells were collected from ovarian follicles 44 hours post-PMSG injection.

### Immunofluorescence staining

Oocytes were collected and fixed with 4% (w/v) paraformaldehyde in PBS (pH 7.4) for 40 min at room temperature. Fixed samples were permeabilized in incubation buffer (0.5% Triton X-100 in 20 mM Hepes, pH 7.4, 50 mM NaCl, 3 mM MgCl_2_, 300 mM sucrose) for 30 min. After washing twice in PBS containing 0.01% Triton-X100, samples were incubated in block solution (PBS containing 1% BSA) for 1 h at RT. The microtubules were localized by incubation for 1 h with a fluorescein isothiocyanate-labeled mouse monoclonal antibody against α-tubulin (Sigma Chemical Co., F-2168), which was diluted 1∶100 in blocking solution. Nuclear statuses of oocytes were evaluated by staining with 10 mg/mL propidium iodide (PI) in PBS for 10 min. Finally, the samples were observed under a Zeiss confocal laser scanning microscope. The area of the spindle was counted using Image ProPlus software.

### Oocyte activation

Oocytes were activated by six-hour treatment with 10 mM SrCl_2_ in Ca^2+^-free CZB medium [19]. Six hours after the activation treatment, oocytes were observed under a microscope. Oocytes were considered as activation when each cell contained one or two well-developed pronucleus or two cells each having one pronuclei.

### *In vitro* fertilization

Collected oocytes were put into G-IVF (fertilization medium, Vitrolife), which was pre-incubated to obtain a steady temperature and humidity (37°C and 95%), and fertilized in the same medium with fresh sperm (obtained from an ICR male donor). *In vitro* fertilization was performed using 1×10^6^/ml motile cauda epididymal sperm, which had been previously capacitated in G-IVF medium for 1 h. Gametes were co-incubated in 50 µl of G-IVF medium covered with mineral oil, for 6 h at 37°C under 5% CO_2_ in air.

### *In vitro* culture

After incubation with sperm for 6 h, oocytes were washed three times in 50 µl drops of G-1 medium (embryo culture medium, Vitrolife). Next, oocytes were placed in 50 µL drops of G-1 medium under oil, and cultured overnight. The embryos at 8-cell stage were transferred to a fresh drop of G-2 medium covered with oil, and cultured for another 48 h. The presence of two polar bodies and pronuclei was determined as the criteria of fertilization.

### TUNEL and confocal microscopy

Cumulus-oocyte complexes (COCs) and oocytes were fixed in 4% (v/v) paraformaldehyde solution for 1 h at room temperature. Membranes were permeabilized in 0.1% Triton X-100 in 0.1% citrate solution for 1 h at room temperature. The TUNEL assay was used to assess the presence of apoptotic cells (*in situ* Cell Death Detection Kit, TMR red; Roche, Mannheim, Germany). Fixed cumulus-oocyte complexes were incubated in TUNEL reaction medium for 1 h at 37°C in the dark. After the reaction was stopped, the COCs were washed three times in PBS. Total cell nuclei were labeled with 10 mg/ml Hoechest 33342 for 5 min in the dark. Slides were stored at −20°C up to 7 days before confocal laser scanning microscopy evaluation. Apoptosis was determined as the percentage of labeled cells to the total cell number.

### Data analysis

Data were analyzed using SPSS 18.0 statistical software. At least three replicates were conducted for each treatment. Data were analyzed using chi-square test and ANOVA, and presented as means ± SD. Statistical significance was determined when *P* value was less than 0.05.

## Results

### Effect of Aroclor 1254 on the number of ovulated oocytes

The average numbers of ovulated oocytes were 25±5, 30±6, and 27±9 after Aroclor 1254 treatment at doses of 12.5, 25 and 50 mg/kg, respectively. There was no significant difference between the control group (28±7) and any of the Aroclor 1254-treated groups.

### Effect of Aroclor 1254 on oocyte parthenogenic activation

Ovulated oocytes were activated in the medium with 10 mM SrCl_2_, the percentage of activated oocytes was decreased with the increase of Aroclor 1254 dose. Table 1 showed that although the percentage of oocyte activation was decreased after 12.5 mg/kg Aroclor 1254 treatment, there were no significant differences compared to the control group. In the 25 and 50 mg/kg Aroclor 1254 groups, the percentages of activated oocytes were significantly lower than that in the control and 12.5 mg/kg Aroclor 1254 groups (Table 1).

**Table 1:** Effect of Aroclor 1254 on the parthenogenetic activation of oocytes.

Group	Total number of ovulated oocytes	Activated oocytes(%)
Control	154	94.8±3.2
12.5 mg/kg Aroclor 1254	132	83.7±2.5
25 mg/kg Aroclor 1254	168	66.6±3.9*
50 mg/kg Aroclor 1254	127	59.4±7.9**

*Compared to control group, *P*<0.05. **Compared to control group, *P*<0.01

### Effect of Aroclor 1254 on spindle morphology of ovulated oocytes

**Figure 1:**
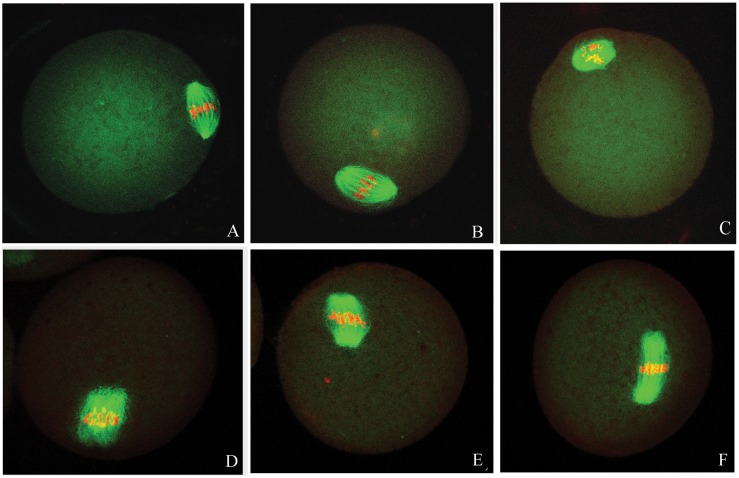
Confocal micrographs of ovulated mouse oocytes treated by Aroclor 1254 showing the morphology of spindle (green) and chromosomes (red). (A) Spindle morphology of ovulated oocytes without treatment of Aroclor 1254 (control). (B–C) *in vivo* matured oocytes were obtained from mouse treated by 12.5, 25 mg/kg Aroclor 1254, respectively. (D–F) oocytes were obtained from mouse treated by 50 mg/kg Aroclor 1254.

**Figure 2:**
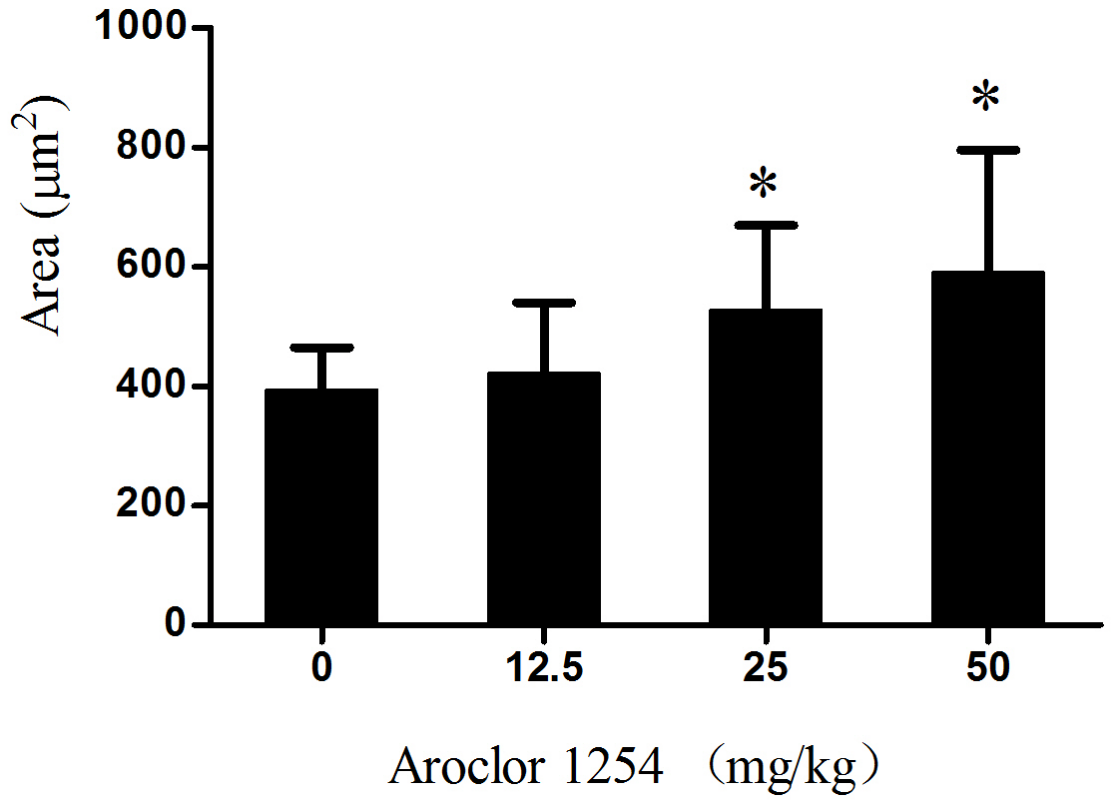
Effects of Aroclor 1254 administration on the spindle area of *in vivo* matured oocytes (*n* = 18). *Compare to control group, *P*<0.05; ^Δ^Compare to 12.5 mg/kg Aroclor 1254 group, *P*<0.05.

**Figure 3:**
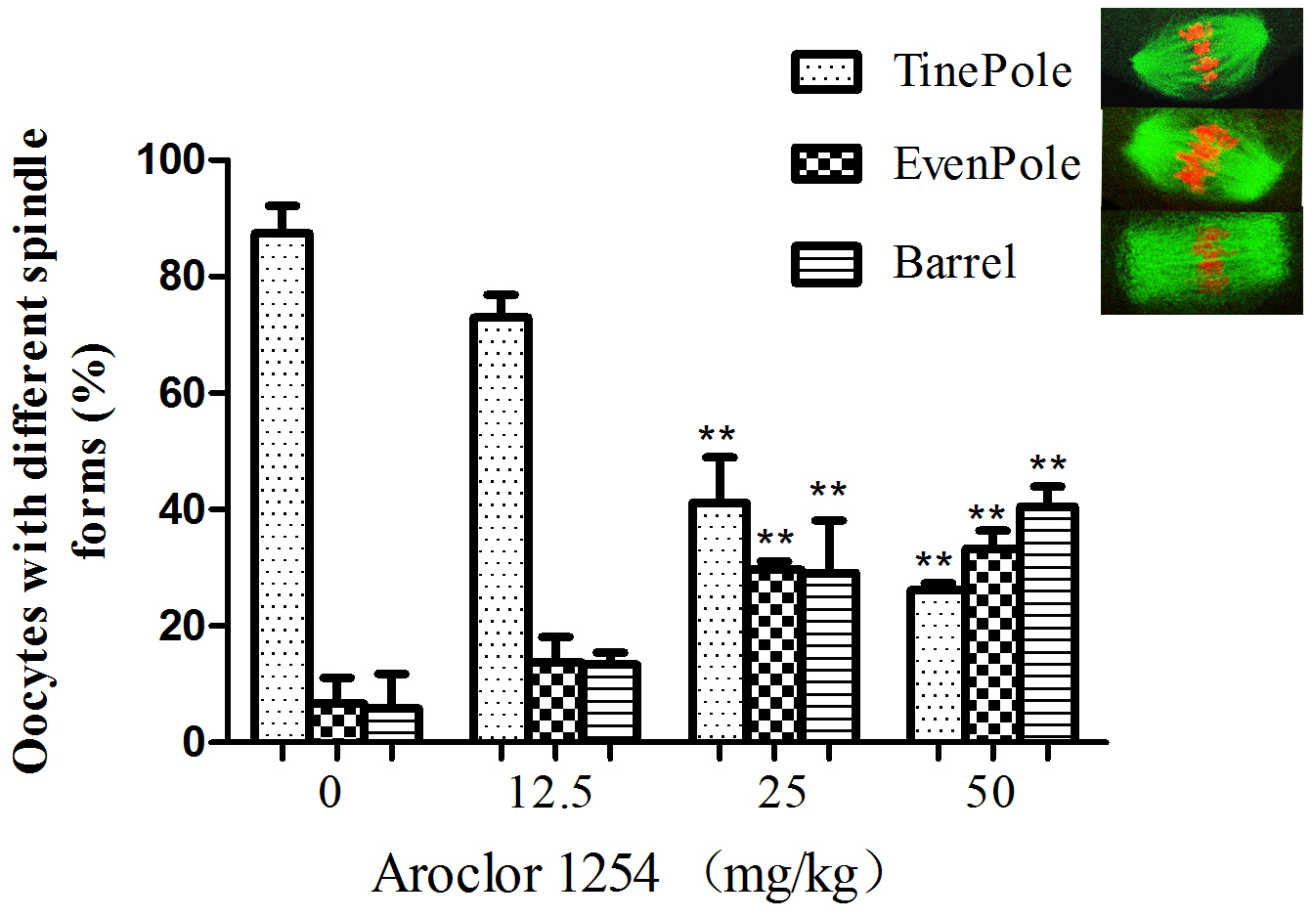
Percentages of oocytes with the tine-pole (TinePole), even-pole (EvenPole), and barrel-shaped (Barrel) spindles under different conditions. **Compare to control group, *P*<0.01.

The ovulated oocytes exhibited bipolar spindles with focused poles in the control and 12.5 mg/kg Aroclor 1254 group (Fig. 1 A, B). In contrast, the oocytes displayed a large barrel configuration in the 25 and 50 mg/kg Aroclor 1254 groups (Fig. 1 C, D, E and F). The spindle areas in the four groups were 393.57±71.26, 422.41±117.51, 527.41±141.77, and 591.14±204.58 µm^2^, respectively (Fig. 2). Spindle area significantly increased with increased Aroclor 1254 dose, demonstrating a dose-dependent relationship. In the 25 and 50 mg/kg Aroclor 1254 groups, the spindle areas were significantly larger than in the control and 12.5 mg/kg Aroclor 1254 groups. Furthermore, chromosomes in oocytes treated by 25 and 50 mg/kg Aroclor 1254 became dispersed (Fig. 1 C, D, E and F). Three spindle types were observed including the tine-pole, even-pole, and barrel-shaped spindles (Fig. 3). While the number of oocytes with barrel-shaped spindles increased, that of oocytes with tine-pole spindles decreased significantly in the 25 and 50 mg/kg Aroclor 1254 groups.

### Effects of Aroclor 1254 on mouse oocyte fertilization and embryo development *in vitro*

To determine if Aroclor 1254 adversely affects oocyte fertilization and embryo development *in vitro*, we performed *in vitro* fertilization and allowed the embryos to develop *in vitro*. Although the percentage of oocytes that fertilized *in vitro* and embryo development was decreased in 12.5 mg/kg treated group, this change had no statistical significance (Table 2). In the 25 and 50 mg/kg Aroclor 1254 groups, the percentages of fertilized oocytes, 4-cell embryos and blastocysts were significantly lower than in the control groups. Aroclor 1254 induced significant injury to fertilization, resulting in inhibition of embryonic development from the zygote to blastocyst stage (Table 2).

**Table 2:** Effects of Aroclor 1254 treatment on mouse fertilization and preimplantation embryonic development.

Group (mg/kg)	No. of oocytes	Fertilization(%)	2-cell(%)	4-cell(%)	Blastocyst(%)
0	255	232 (91.3±5.7)	218 (85.0±6.6)	203 (78.7±6.9)	143 (57.4±6.7)
12.5	184	150 (84.5±10.8)	141 (76.5±6.5)	136 (74.0±1.9)	95 (51.7±3.2)
25	259	212 (81.2±2.0)**	189 (70.1±8.0)**	169 (64.7±7.4)**	89 (39.9±16.0)**
50	224	174 (76.4±11.9)**	132 (62.0±10.9)**	109 (49.3±6.1)**	59 (30.5±15.2)**

* Compare to control group, *P*<0.05; ** Compared to control group, *P*<0.01. Figures in parentheses indicate percentage.

### Effects of Aroclor 1254 on the incidence of apoptosis in cumulus cells

**Figure 4:**
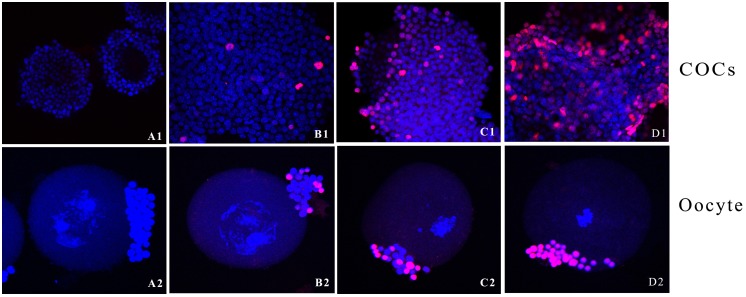
Representative images of cumulus-oocyte complexes and oocytes after exposure to Aroclor 1254 subjected to TUNEL analysis. COCs and oocytes representative samples from (A1 and A2) the control group, (B1 and B2) the 12.5 mg/kg Aroclor 1254-treated group, (C1 and C2) the 25 mg/kg Aroclor 1254-treated group, and (D1 and D2) the 50 mg/kg Aroclor 1254-treated group for TUNEL analysis are shown. Red staining indicates fragmented DNA in cumulus cells undergoing apoptosis, whereas intact cell nuclei are stained blue respectively.

**Figure 5:**
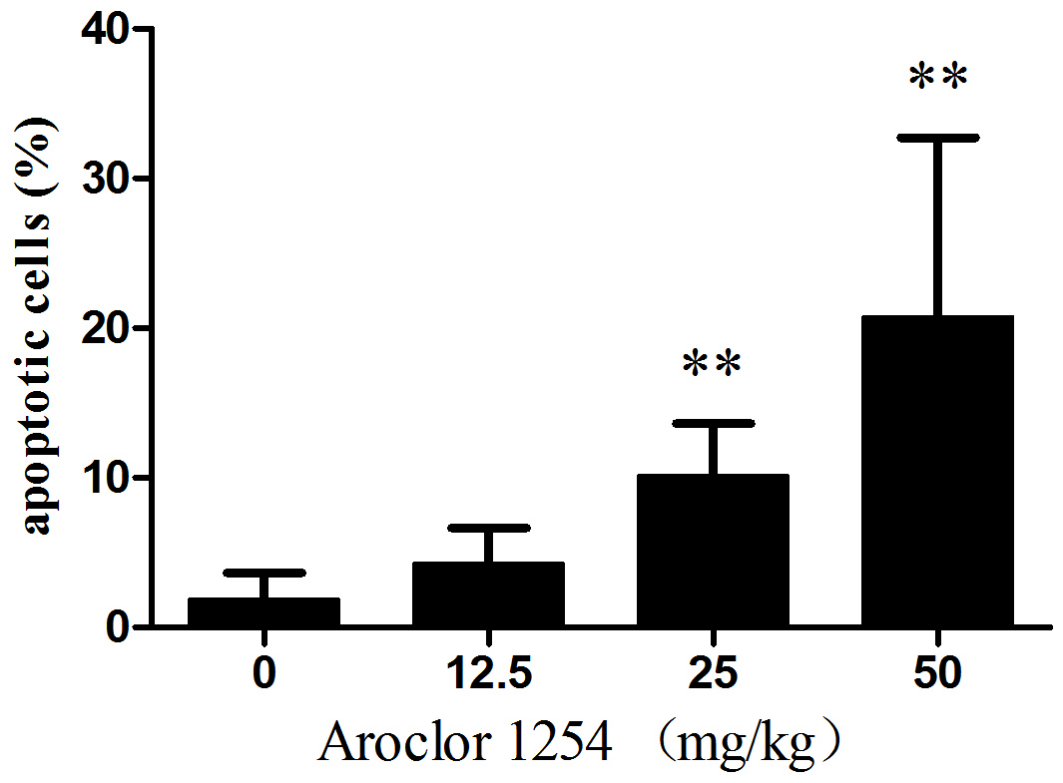
Effects of Aroclor 1254 exposure on apoptosis incidence in mouse cumulus-oocyte complexes. ** Compare to control group, *P*<0.01.

As shown in Fig. 4 A1–D1 and Fig. 5, the incidence of apoptosis measured by the TUNEL assay in COCs treated with 25 and 50 mg/ml Aroclor 1254 was significantly higher compared with controls. Fig. 4 shows that although the rate of apoptosis was decreased after 12.5 mg/kg Aroclor 1254 treatment, there were no significant differences compared to the control group. Furthermore, chromatin in denuded oocytes treated by 25 and 50 mg/kg Aroclor 1254 became agglomerated (Fig. 4 A2–D2).

## Discussion

There is much concern about exposure to Aroclor 1254 causing toxic effects including reproductive toxicity in humans and animals. Numerous studies have shown that Aroclor 1254 exposure causes subchronic and acute damage to manmals, likewise reproductive toxicity [11], [20]. Furthermore, most data available are from *in vitro* models. There are large differences between *in vitro* and *in vivo* models [21]. The latter requires further attention, especially on the effects of Aroclor 1254 on mammalian oocytes *in vivo* [14]. Our present study revealed that oocytes exposed to Aroclor 1254 *in vivo* were characterized by reduced developmental potential, presented abnormal chromosomal arrangements and abnormal spindle configurations.

Aroclor 1254 inhibited in the vitro maturation and development competence of bovine oocytes [22] as well as inducing cumulus cell apoptosis [23]. Furthermore, adverse effects of exposure to Aroclor 1254 during oocyte maturation were extended to fertilization and embryonic development [8]. To further study the effect of Aroclor 1254 on reproductive function, we investigated the effects of Aroclor 1254 on mammalian oocyte outgrowth potential *in vivo*.

Aroclor 1254 doses that correspond to 12.5, 25, 50 mg/kg (much lower than the experimental LD_50_ dose) were administered to female mice by intraperitoneal injection. The purpose was to inspect whether low doses exposed to Aroclor 1254 could adversely affect the maturation and developmental competence of mouse oocytes *in vivo*. In order to prove the reproductive system sensitivity to PCBs compared to the immune system and the nervous system, lower PCBs concentration were used in this experiment [24], [25]. Here, we examined the effect of hypodermic exposure to Aroclor 1254, at a concentration similar to that found in the environment [16], [26], [27], on female gametes of mice. Pauwels et al. reported that PCBs could be found in the follicular fluid and the concentrations ranged from nanograms to micrograms per ml [28]. PCB 153, which is a commonly detected PCB congener in biological tissues [29], [30], [31] and milk [32], accounted for about 5.64% in Aroclor 1254 [33]. The concentration of PCB 153 in plasma of rats was 9 ng/g lipids after treated with 25 mg/kg Aroclor 1254 for three times by Bergman [16]. The concentration of PCB 153 was 500 ng/g lipid in follicular fluid collected from a fertility clinic in Rome [34]. The concentration of PCB 153 in mother's milk in Latvia was 16.150 ng/g lipids [35]. Although a considerable variation in the relative amounts of the different PCBs is observed in different species, 9 ng/g is less than 500 ng/g and 16.15 ng/g. The drug concentration in follicular fluid of our experimental design may much lower than the environmental, which is reasonable and meaningful.

Our results showed that Aroclor 1254 doses of 25 and 50 mg/kg decreased the percentage of parthenogenetically activated oocytes significantly, by causing larger abnormal spindles. Furthermore, these concentrations appear to be an ample margin of safety for human oocytes development at the present exposure levels. However, possible cumulative exposure to other contaminants and xenobiotics with similar mechanisms of toxicity or possible storage of these compounds in body fat could result in a clinically important effect of Aroclor 1254 exposure.

Strontium has been used often to activate oocytes for analytical studies of oocyte quality [36], [37] and activation [19]. Oocytes are activated by a series of Ca^2+^ oscillations [38]. In this paper, medium contained strontium chloride was used to stimulate intracellular Ca^2+^ release that induces Ca^2+^-dependent biological responses, and generates parthenogenetic activation. A previous study found that the effects of PCBs on neutrophil function can be explained by effects on Ca^2+^/calmodulin-dependent action [39]. In consideration of the decreased parthenogenetic activation in the 25 and 50 mg/kg Aroclor 1254 groups, we suspect that Aroclor 1254 may potentially affect intracellular Ca^2+^ release or alter Ca^2+^ concentration in the oocyte cytoplasm. In addition, the repressive effect on the parthenogenetic activation of oocytes was consistent with the adverse effect on spindle area in the 25 and 50 mg/kg Aroclor 1254 groups. We hypothesized that the subsequent fertilization and embryo development process would be impacted by Aroclor 1254, so further studies were done.

The effect of compounds on oocyte maturation could be evaluated not only on the nuclear maturation but also on the cytoplasmic maturation. The cytoplasmic maturation could be evaluated by the early embryo development [40]. In the 25 and 50 mg/kg Aroclor 1254 groups, the fertilization and embryo development rates were significantly lower than in the control group, which means oocytes exposed to Aroclor 1254 *in vivo* were characterized by reduced development potential, as evident from decreased oocyte cleavage to the blastocyst stages. These data are in agreement with the observations of Meeker JD et al. [41], which revealed reduced fecundity and increased time to pregnancy (TTP) among women exposed to PCBs.

Mechanisms underlying this adverse effect have not yet to be elucidated. Recently, spindle analysis was used to assess oocyte quality [42] and the effects of toxicants and drugs on oocytes [36], [37]. Spindle formation was considered the indicator of oocyte maturation. Meiotic spindles are determinant to the normal chromosome alignment and the separation of chromosomes during the maturation of oocyte [43], [44]. The normal MII spindle has a characteristic spindle shape. The present study showed that *in vivo* Aroclor 1254 treatment induced abnormal, larger-barrel spindles. These data are in agreement with the observations of Brevini et al. [45], which demonstrated that exposure to PCBs mixtures caused a perturbation of the assembly of a microtubule cytoplasmic network during porcine oocyte maturation. Though the mechanism of spindle injury is not yet clear, mitochondria may be involved in the regulation of microtubule dynamics [46]. A disruption of mitochondrial aggregation during oocytes maturation after PCBs treatment was observed [45], [47]. These data suggest that Aroclor 1254 exposure is likely to induce a generalized impairment of the mammalian oocyte organelles. Although, our results are the first to report the abnormal spindle configurations induced by exposure to Aroclor 1254, further evidence is needed to better elucidate this aspect.

The mammalian oocyte and its surrounding cumulus cells are interdependent throughout the growth and development of the oocyte and ovarian follicle. The function of cumulus cells is to protect the growing oocyte from the disadvantageous environment and to supply nutrients to oocyte through gap junctions between the cumulus mass and the oocyte [48]. Granular cells play a crucial role in regulating oocyte maturation and fertilization, because of their close proximity to the oocyte [49], [50]. Although apoptosis was not found in oocytes, chromatin in oocytes treated by Aroclor 1254 became agglomerated. Our data showed that the levels of apoptosis in cumulus cells are increased after exposure to Aroclor 1254 *in vivo*. Taken together, the presence of apoptosis in cumulus cells and the poor outgrowth potential in oocytes may also indicate a specific role of the cumulus cells in mediating Aroclor 1254 toxicity during oocyte maturation. It is possible to conjecture that cumulus cells may be target cells for toxicological injury induced by Aroclor 1254 during oocyte maturation.

In conclusion, we found that Aroclor 1254 treatment *in vivo* induced abnormal spindle configurations and affected the normal development process of oocytes. Aroclor 1254 also induced significant injury to oocyte fertilization, resulting in inhibition of embryonic development from the zygote to blastocyst stage. Our data points to increased levels of apoptosis in cumulus cells may account for the reduced developmental potential of Aroclor 1254-treated oocytes. Aroclor 1254 toxicity to reproductive function deserves further investigation. It is clear that Aroclor 1254 affects oocyte outgrowth potential through a wide variety of biological processes and molecular mechanisms. These findings may explain previous reports of reduced fecundity among women exposed to PCBs.
